# Molecular defects of the GNRH receptor gene in Chinese patients with idiopathic hypogonadotropic hypogonadism and the severity of hypogonadism

**DOI:** 10.1186/1687-9856-2013-S1-O25

**Published:** 2013-10-03

**Authors:** Aws K Fathi, Sicui Hu, Xi Fu, Shan Huang, Yan Liang, Xiaoping Luo

**Affiliations:** 1 Department of Pediatrics, Tongji Hospital, Tongji Medical College, Huazhong University of Science and Technology, Wuhan 430030, China

## 

To identify and determine the frequency of mutations in the coding region of the gonadotropin-releasing hormone receptor (GnRHR) gene in forty Chinese patients with normosmic idiopathic hypogonadotropic hypogonadism (IHH) and establish genotype/phenotype correlations where possible.

The diagnosis of HH was based on absent or incomplete sexual development after 17 yr of age in girls and 18 yr in boys associated with low or normal levels of LH in both sexes and low levels of testosterone in males and of estradiol in females. All patients presented with a normal sense of smell in an olfactory specific test.40 IHH patients and 40 controls were screened for mutations in the coding sequence of GnRHR gene. The coding region of the GnRHR gene was amplified by PCR and directly sequenced.

A missense mutation serine168arginine (S168R) located in the fourth transmembrane domain of the GnRH-R gene was identified in a homozygous state in one male with complete HH, the S168R mutation has been previously

Shown to cause in the complete loss of receptor function because hormone binding to the receptor is completely impaired. In another patient, a compound heterozygous mutation (Gln106Arg and Arg262Gln) was identified in a male with partial HH, the Gln106Arg mutation located in the first extracellular loop of GnRH-R, this mutation decrease but not eliminate GnRH binding, while Arg262Gln mutation located in the third extracellular loop of GnRH-R and only decreases signal transduction.

A good correlation between genotype and phenotype was found in our patients. The patient, who is homozygous for the completely inactivating S168R mutation, has complete HH. In addition, the affected patient who is compound heterozygotes for the Gln106Arg - Arg262Gln mutations, has partial HH.

GnRHR mutations can be classified into partial or complete loss of function mutations. Partially inactivating substitutions of the GnRHR frequently found in familial hypogonadotrophic hypogonadism are Q106R and R262Q. comparison of compound heterozygous with homozygous patients suggests that their phenotype and the response to GnRH is determined by the GnRHR variant with the less severe loss of function.

